# Comparative Transcriptome Profiling of *Kappaphycus alvarezii* (Rhodophyta, Solieriaceae) in Response to Light of Different Wavelengths and Carbon Dioxide Enrichment

**DOI:** 10.3390/plants10061236

**Published:** 2021-06-17

**Authors:** Vun Yee Thien, Kenneth Francis Rodrigues, Christopher Lok Yung Voo, Clemente Michael Vui Ling Wong, Wilson Thau Lym Yong

**Affiliations:** 1 Biotechnology Research Institute, Universiti Malaysia Sabah, Jalan UMS, Kota Kinabalu 88400, Malaysia; vunyee.thien@xmu.edu.my (V.Y.T.); kennethr@ums.edu.my (K.F.R.); cvooly@ums.edu.my (C.L.Y.V.); michaelw@ums.edu.my (C.M.V.L.W.); 2 Innovation Center, Xiamen University Malaysia, Jalan Sunsuria, Bandar Sunsuria, Sepang 43900, Malaysia; 3 Seaweed Research Unit, Faculty of Science and Natural Resources, Universiti Malaysia Sabah, Jalan UMS, Kota Kinabalu 88400, Malaysia

**Keywords:** gene expression, light regulation, macroalgae, photosynthesis, transcriptome sequencing

## Abstract

Rhodophyta (red algae) comprises over 6000 species, however, there have only been a few comparative transcriptomic studies due to their under-representation in genomic databases. *Kappaphycus alvarezii*, a Gigartinales algae, is a valuable source of carrageenan and is extensively cultivated in many countries. The majority of seaweed farming in Southeast Asia is done in intertidal zones under varying light (i.e., spectra and irradiance) and carbon dioxide (CO_2_) conditions, which affects the rate of photosynthesis. This study conducted transcriptome profiling to investigate the photosynthetic mechanisms in *K. alvarezii* exposed to different wavelengths of light (i.e., blue, green, and red light, in comparison to white light) and CO_2_ availability. We analyzed the responses of photosynthetic protein complexes to light and observed that light of different wavelengths regulates a similar set of photosynthetic apparatuses. Under CO_2_ enrichment, genes encoding C_3_ and C_4_ enzymes were found to be actively transcribed, suggesting the likely shift in the carbon metabolism pathway or the involvement of these genes in adaptive physiological processes. This study contributes to the understanding of the regulatory mechanisms of photosynthetic carbon metabolism in red algae and has implications for the culture and commercial production of these economically valuable macroalgae.

## 1. Introduction

Transcriptome shifts associated with the light signals perceived by photoreceptors offer insights into the gene regulation in higher plants; however, limited information is available on seaweed exposed to different wavelengths of light. The red alga, *Kappaphycus alvarezii* (Doty) Doty, is the most cultivated seaweed in the world, and its commercial farms are usually placed in shallow water areas in the intertidal zones [1,2]. Seaweed grown in the intertidal zone may face depletion in both quality and quantity of light with the changes in spectral distribution at different depths in the water column [3]. However, seaweeds can acclimate to the changes in light intensity and wavelength by employing efficient light-harvesting mechanisms, such as increasing the quantum of photosynthetic pigments or changing the ratio of accessory pigments to chlorophyll *a* [4,5]. Light-mediated physiological responses and molecular mechanisms have been studied in brown alga, *Saccharina japonica*, whose gene expression profiling indicated that light induces significant changes in gene expression in the species [6,7]. These light-responsive genes encode many transcription factors, particularly those involved in various functions such as photomorphogenesis, circadian clock function, photoreactivation, pigment biosynthesis, and carbon fixation.

The transcriptome of *K. alvarezii* was first sequenced by Wu et al. [8] together with that of 19 Phaeophyceae (brown algae) and other 20 Rhodophyceae (red algae). Their study provided a broad range of algal transcriptome data of potential genetic and biological studies linking the genes to their functions. Then, Song et al. [9] performed a de novo transcriptome sequencing of *K. alvarezii*, along with other species of the Solieriaceae family (e.g., *Betaphycus gelatinus* and *Eucheuma denticulatum*) to investigate the mechanisms underlying carrageenan biosynthesis. A recent transcriptome profiling of *K. alvarezii* elucidated further genetic information and revealed genes involved in some important metabolic pathways such as energy metabolism, carbohydrate metabolism, and amino acid metabolism that maintain the essential functions of the organism [10]. To date, very few studies have elucidated the molecular mechanism of photosynthesis in *K. alvarezii* [11]. The occurrence of different pathways and photosystem complexes in plants may have resulted due to the exposure to different environmental conditions, e.g., light regimes and temperature [12,13]; besides, the alterations in photosynthetic pathways under environmental changes possibly contribute to the adaptation of plants to environmental stresses [14,15]. While the C_3_ pathway is predominantly active in marine seaweed [16,17,18], C_4_ photosynthesis has also been observed in some other eukaryotic algae [19,20]. Furthermore, the coexistence of C_3_ and C_4_ photosynthetic pathways have been observed in *Ulva prolifera* and other aquatic plants, including *Chara contraria*, suggesting that these species may alter their carbon metabolism pathways in response to environmental changes, or may carry out both C_3_ and C_4_ cycles under normal conditions, with a possibly more significant role of C_4_ photosynthesis under stress conditions [21,22].

Seaweeds have developed mechanisms to regulate their photosynthetic activity and adapt to the changing environments, including the light regime, temperatures, CO_2_, and nutrient levels [23,24,25]. Photosynthetic regulation by light quality involves chloroplast structure, pigment composition, antioxidant defenses, and photosynthetic carbon assimilation [26,27,28]. Unlike land plants, seaweeds can synthesize pigments complementary to the spectral quality of the incident light [29]. The process of harvesting light in red algae is carried out primarily by a group of pigmented proteins, called phycobiliproteins, that are constituents of a macromolecular complex called the phycobilisome [11,30]. On the other hand, photosynthesis in many seaweeds is nearly saturated with the ambient dissolved inorganic carbon because of their capacity to utilize a high concentration of seawater bicarbonate (HCO_3_^−^) as the principal inorganic carbon source [31]. Although the seaweeds exhibit wide-ranging capacities to extract HCO_3_^−^ from the seawater, they often show heterogeneous and species-specific responses to elevated CO_2_ [32,33].

In this study, we used Illumina sequencing technology for transcriptome profiling of *K. alvarezii* to determine a possible differential gene expression in response to light of different wavelengths and CO_2_ enrichment. The detailed analysis was performed by comparing the transcriptomes of *K. alvarezii* exposed to white light (WL) and the three other wavelengths, i.e., blue (BL), green (GL), and red light (RL), and between with and without CO_2_ enrichment. The transcriptome dataset was also compared with those of red algae deposited earlier in the GenBank [8], and a significant similarity was observed between the current and the earlier transcriptomes. This information adds to our understanding of molecular mechanisms underlying light-induced responses, adaptation, and inorganic carbon fixation in red algae, and it provides novel insights into the impact of changing light conditions on *K. alvarezii* growth and propagation.

## 2. Results

### 2.1. Transcriptome Sequencing and De Novo Assembly

The sequencing run generated a total of 31.89 Gb raw paired-end reads from all the RNA samples across all treatments. After the identification and removal of adapters and low-quality reads, over 25 Gb filtered reads (≈79%) were retained for assembly and further analysis. Using Trinity, a total of 76,871 assembled transcripts were generated with lengths in the range of 300 to 17,257 bp with a mean length of 979 bp and an N50 contig length of 1707 bp. There were no ambiguous bases within the assembled sequences. In the quality assessment of the assembly, most of the reads (93.51%) aligned back to the assembled transcripts with an average read depth of 256.47. The reads that did not map back to the assembled transcripts corresponded either to shorter than 300 bp or with incomplete/unmatched sequences with the transcripts.

### 2.2. Functional Anotation of the K. alvarezii Transcriptome

**Figure 1:**
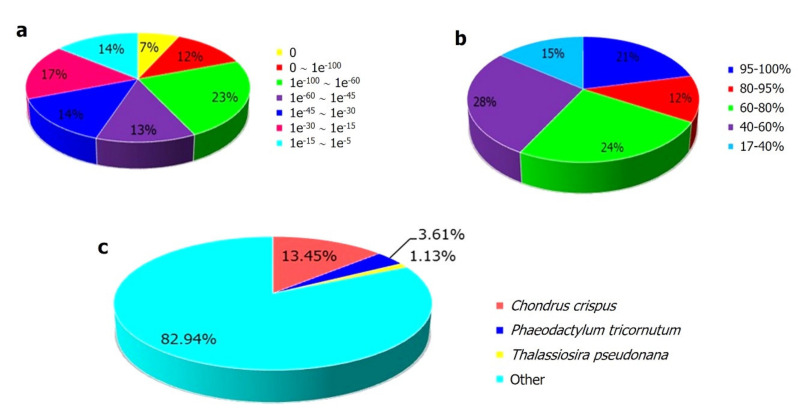
Functional annotation of the *Kappaphycus alvarezii* transcriptome. Characteristics of the similarity search of the assembled sequences against the Uniprot databases. (**a**) *E*-value distribution of BLAST hits for each unique sequence with *E*-value ≤ 10^−5^. (**b**) Similarity distribution of the top BLAST hits for each sequence. (**c**) Species distribution of total homologous sequences with *E*-value ≤ 10^−5^. The first hit of each sequence was used for statistical analysis.

A total of 42,915 transcripts could be assigned with putative functions, accounting for 55.83% of the total assembled sequences. UniProt annotation revealed that 23,701 (55.23%) transcripts had high-confidence hits of *E*-value smaller than 10^−45^ (Figure 1a), and 21.42% of the transcripts had over 95% similarity (Figure 1b). In terms of species distribution, the top hit result showed that only 13.45% of the transcripts matched with those of the red alga *Chondrus crispus*, which was followed by 3.61% of matches with those of the diatom *Phaeodactylum tricornutum* (Figure 1c).

**Figure 2:**
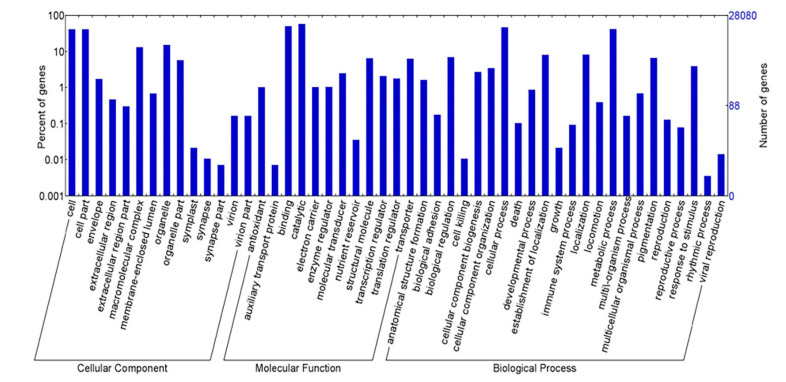
Gene Ontology (GO) annotation of non-redundant transcripts of *Kappaphycus alvarezii*. All the transcripts were grouped into three major functional categories, as indicated.

About 28,079 annotated transcripts were grouped into three main GO domains, i.e., 20,847 sequences (27.13%) into “biological process”, 14,291 (18.60%) into “cellular component”, and 41,698 (54.27%) into “molecular function” (Figure 2). Functional and pathway analysis assigned a total of 10,460 transcripts to 278 KEGG pathways and the largest numbers of transcripts were assigned to “metabolic pathways”, followed by the “biosynthesis of secondary metabolites”, “biosynthesis of amino acids”, and “carbon metabolism” (Table 1).

### 2.3. Identification of Candidate Transcripts Associated with light and CO_2_ Responses

Based on sequence similarity, several transcripts associated with the response to light were identified, among which 72 candidates were of photosynthetic protein complexes, i.e., chlorophyll, carotenoids (beta- and zeta-carotene), phycobilisome, phycocyanin, and phycoerythrin, and 38 candidates were of photosystem proteins, including (i) photosystem I (PSI) subunits (PsaA, PsaB, and PsaC), PSI chlorophyll A apoprotein, Ycf3 and Ycf4 protein domains; (ii) photosystem II (PSII) subunits (D1, D2, CP43, and CP47), PSII extrinsic protein PsbP, and PSII reaction center protein M. Out of the 38 photosystem proteins, 15 were PSI and 23 were PSII proteins. Three types of photoreceptor candidates, including one cryptochrome, two blue-light receptors (PHR2), and four phytochrome proteins were also identified. One phytochrome protein was found to be similar to the phytochrome-like protein 2, which is a photoreceptor that exists in two forms that are reversibly interconvertible by light [34]. We found 307 candidates of the protein kinase (PK) family, including serine/threonine PK, calcium-dependent PK, and receptor-like PK in the light-associated transcripts; a total of 59.93% PKs in *K. alvarezii* were homologous to those in *C. crispus*. Furthermore, candidate genes associated with both C_3_ and C_4_ photosynthetic pathways were found in *K. alvarezii*, and a total of 129 carbon fixation-related transcripts in response to CO_2_ levels were identified. The transcripts of key enzymes, such as phosphoribulokinase, phosphoglycerate kinase, aspartate aminotransferase, phosphoenolpyruvate carboxylase, phosphoenolpyruvate carboxykinase, malate dehydrogenase, and pyruvate orthophosphate dikinase were also identified (Table 2) and provided unequivocal molecular evidence that both the C_3_ and C_4_ pathway genes are actively transcribed in *K. alvarezii*.

### 2.4. Identification of Differentially Expressed Genes (DEGs)

**Figure 3:**
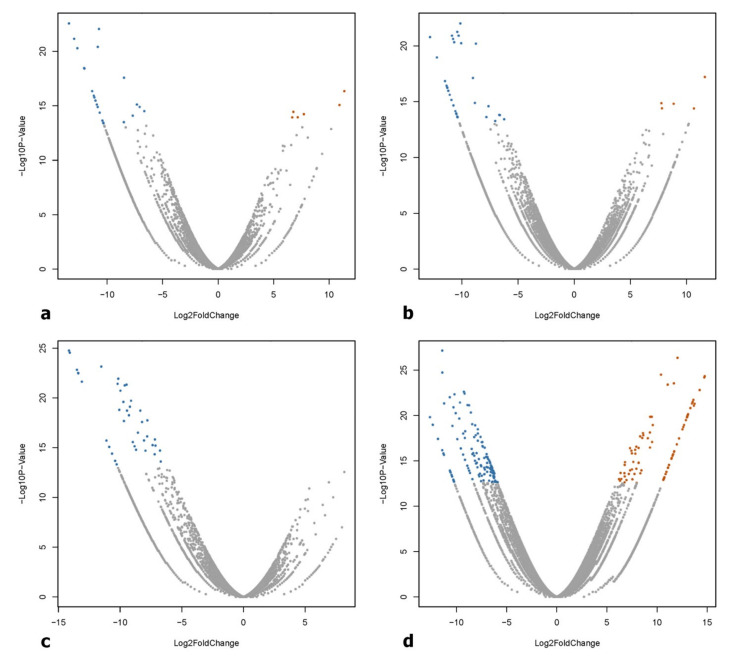
Volcano plots of differentially expressed transcripts (red indicates upregulation and blue indicates downregulation) between (**a**) WL and BL, (**b**) WL and GL, (**c**) WL and RL, and (**d**) without and with CO_2_ enrichment, at an adjusted *p*-value < 1 × 10^−10^ and a log_2_ fold change ≥ 2. WL, white light; BL, blue light; GL, green light; RL, red light.

**Figure 4:**
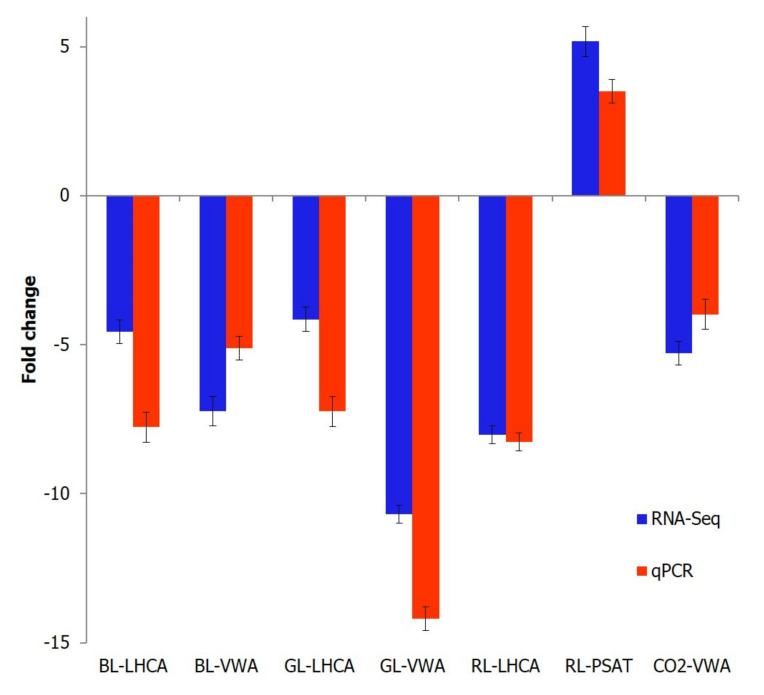
A comparison of differential gene expressions in *Kappaphycus alvarezii* by RNA-Seq and real-time PCR. LHCA, light-harvesting complexes; VWA, Von Willebrand factor type A; PSAT, phosphoserine aminotransferase; BL, blue light; GL, green light; RL, red light.

The high-quality reads (84 to 86%) from individual samples were mapped to the transcriptome database and deposited in the National Center for Biotechnology Information (NCBI) Sequence Read Archive (SRA) with the following accession numbers: SRR2757332 (GL), SRR2757333 (BL), SRR2757334 (CO_2_-treated), SRR2757335 (WL), and SRR2757337 (RL). The lower mapping rate of the reads to existing libraries reflects the low transcriptome sequencing depth and a high level of heterozygosity in *K. alvarezii*. Gene expression levels in *K. alvarezii* exposed to different light wavelengths and CO_2_ enrichment were compared in pairs and groups of treatments. A total of 1487 DEGs were detected using stringent filtering criteria, such as an adjusted *p*-value < 1 × 10^−10^ and a log_2_ fold change of two as the cutoff (Figure 3). The results were validated by qPCR of the selected genes (Table 3), including two genes in each of BL (LHCA and VWA), GL (LHCA and VWA), and RL (LHCA and PSAT), and one gene in the CO_2_-induced condition (VWA), confirming the accuracy of RNA-Seq in mRNA quantification (Figure 4). Of the 1311 classified DEGs, 931 (69.64%) were then annotated successfully through sequence similarity searches against the UniProt database with a significance of *E*-value ≤ 10^−5^. However, about 49.29% of the annotated DEGs could not be assigned to available protein databases and are likely to represent uncharacterized proteins due to the limited availability of sequence data in the databases.

**Figure 5:**
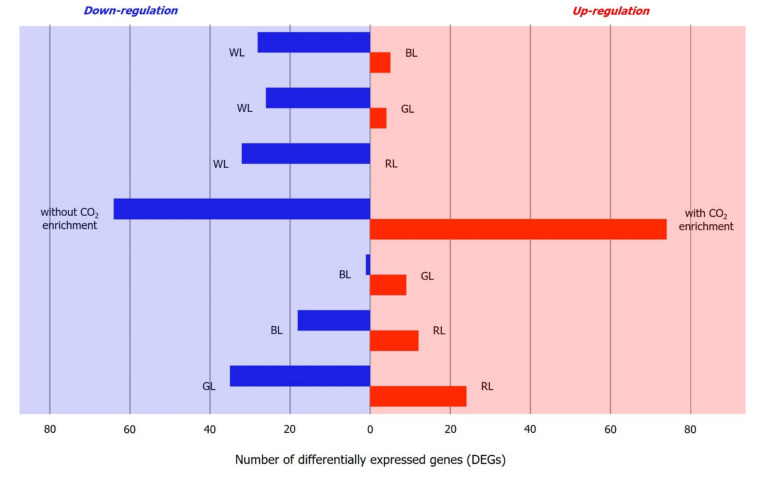
Total numbers of upregulated (red) and downregulated (blue) genes in *Kappaphycus alvarezii* between two treatments (with and without CO_2_ treatment and between different light treatments) with an adjusted *p*-value < 1 × 10^−10^ and a log_2_ fold change ≥ 2. BL, blue light; GL, green light; RL, red light.

A comparison of different light wavelengths against WL revealed the largest number of DEGs in BL-treated samples, with five upregulated and 28 downregulated DEGs (Figure 5). In the CO_2_ enrichment experiment, a total of 138 transcripts were found differentially expressed, with 74 upregulated and 64 downregulated transcripts (Table 4). Overall, the highest number of DEGs were detected between WL and the composite of BL+GL+RL and the highest number of significantly up/downregulated genes encoded ribosomal proteins, indicating a high turnover rate of protein synthesis. Furthermore, a majority (578/582) of the transcripts were found downregulated, implying that these may be associated with differential intensities of the composite and individual wavelengths. Pathway enrichment analysis revealed that the annotated DEGs under different light and CO_2_ treatments were mainly involved in “metabolic pathways”, “biosynthesis of secondary metabolites”, “oxidative phosphorylation”, “carbon metabolism”, and “porphyrin and chlorophyll metabolism”.

## 3. Discussion

Seaweeds grown in the intertidal zones are exposed to fluctuating environmental conditions and have thus developed specific mechanisms enabling them to overcome unfavorable disturbances and environmental stresses [35,36,37]. These adaptations and acclimation responses contribute significantly to their survival and fitness, providing them with the plasticity necessary for a stationary life. The present study enhances our understanding of the gene expressions and responses of red alga *K. alvarezii* toward different wavelengths of light (BL, GL, RL, and WL) and increasing CO_2_ levels.

The reason for the presence of a large number of unknown coding sequences (CDS) in this study can be attributed to the dearth of information about the red seaweed genome [38,39,40]. Alternatively, the number of CDS is likely to be the result of eukaryotic symbionts associated with the seaweed samples [41,42]. However, in this study, we only reported the genes that could be mapped back to the UniProt protein database for red algae and validated their expression using qPCR. For accurate and reliable gene expression results, normalization of real-time PCR data against an internal control gene is required; an ideal control gene should have consistent expression regardless of the experimental treatments and developmental stages of the studied organism [43,44,45,46]. As applied earlier in studies on other red algae and plant species [47,48,49], rDNA genes were used in this expression study for copy number validation because the copy number of the conventional markers such as glyceraldehyde-3-phosphate dehydrogenase and beta-tubulin were not constant across samples as per the results obtained by quantitative reverse transcription PCR.

Our analysis revealed that cDNA synthesized by reverse transcription using oligo(dT) priming includes not only sequences from nuclear mRNA but also transcripts derived from genes encoded on the plastid genome. This result is consistent with the previous findings on polyadenylation of chloroplast mRNAs in the green alga *Chlamydomonas reinhardtii* [50,51,52], flagellate *Euglena gracilis* [53], and higher plants [54,55]. The phenomenon of post-transcriptional modification of mRNA via polyadenylation of the 3′ end has been reported across the plant kingdom [56]. Therefore, we can only concur that plastid-encoded genes are polyadenylated based on the selection of our protocols. However, the future investigator may consider the use of oligo(dT) primer along with a pair of gene-specific primers to validate this phenomenon.

Photosynthesis begins with the process of light harvesting, which is mediated by pigment-binding proteins that form light-harvesting antenna systems. Light-harvesting complexes (LHCs) in red algae constitute a large family of proteins, including chlorophyll *a*, zeaxanthin, and *β*-carotene [57,58]. Chlorophyll *a*-binding LHCs in red algae are functionally associated with PSI and have similarities with higher-plant chlorophyll *a/b*-binding proteins and fucoxanthin chlorophyll *a/c*-binding antenna complexes, as observed in their amino acid sequences and conserved regions [59,60]. Chlorophyll *a/b*-binding and fucoxanthin chlorophyll *a/c*-binding proteins were frequently reported to be transcriptionally repressed in response to light stress in terrestrial plants and microalgae [61,62,63]. In this study, several genes encoding light-harvesting complex A (LHCA) proteins were significantly downregulated after treatment with light of different wavelengths (BL, GL, and RL) in comparison to WL. Previous transcriptomic studies of stress responses also revealed the upregulation of fucoxanthin chlorophyll *a/c*-binding proteins in response to heat-, salt-, and oxidative stress, apart from light in brown algae [64,65], indicating possible additional functions of these proteins. Recently discovered “red lineage chlorophyll *a/b*-binding-like proteins” (RedCAPs) were found to be restricted to the red algal lineage and participate in the light (intensity- and quality-) dependent structural remodeling of light-harvesting antennae [66]. However, the existence and expression of RedCAPs in response to different light qualities and CO_2_ levels in *K. alvarezii* remains to be explored.

Chlorophyll and carotenoids in red algae do not function as primary light absorbers, as the role is taken over by the phycobilisomes, which direct the energy to the reaction center of PSII for conversion into chemical forms [57]. The phycobilisome protein complex consists of two or three types of pigment proteins known as biliproteins, i.e., phycoerythrin, phycocyanin, and allophycocyanin. They differ in protein identity, chromophore type, attachment, and their relative location in the phycobilisome complex [67]. The phycobilisome absorbs light in the 590–650 nm region of the solar spectrum, which cannot be absorbed either by chlorophyll or carotenoids. Thus, organisms with phycobilisomes have greater accessibility to usable light within the visible spectrum and hence possess greater adaptability and light-capturing capacity [67]. Although RL had a significant impact (*p* < 0.05) on the growth of *K. alvarezii* (8.1 ± 1.4% day^−1^) compared to those treated with BL (3.5 ± 1.2% day^−1^) and GL (5.2 ± 1.1% day^−1^), as demonstrated in a related study conducted in our laboratory [68], different light spectra seemed to induce the same effect on phycobilisome content in this species, and the genes encoding phycobilisomes showed no significant difference in expressions when treated with the four wavelengths of light. Conversely, previous studies reported the influence of both irradiance and spectral composition on the synthesis of phycoerythrin and phycocyanin in the red algae [69,70,71,72]. Such differences may be because the differential expression analysis with transcriptome data considers the factors that could vary between samples, including overall depth of sequencing, read and fragment length, gene density in the genome, transcriptome splicing complexity, and transcript abundance [73]. This study was conducted over a duration of 14 days, which may not be sufficient to study long-term changes in gene expression related to phycobilisome biosynthesis. Furthermore, the experimental design did not account for relative protein concentrations, so we can only make inferences based on the number of transcripts, all of which may not be translated into functional proteins. On the other hand, expressions of genes associated with phycobilisome, phycoerythrin, and photosystem proteins were found upregulated in *K. alvarezii* under CO_2_ enrichment, suggesting that the photosynthetic efficiency of this species may be enhanced by increasing CO_2_ levels.

Photosystems are functional and structural units of protein complexes involved in photosynthesis, but there was no significant difference in the expressions of these protein complexes under four different wavelengths of light in *K. alvarezii*. Light intensity and quality are essential factors for photosynthetic competence, and generally higher pigment content is found in algae grown at lower light intensity [74,75,76,77]. While acclimation to high light intensity is shown by reducing phycoerythrin content in phycobilisomes, red algae do not undergo complementary chromatic adaptation and exhibit significant changes in the phycoerythrin/phycocyanin ratio when grown under different wavelengths of light [57]. However, in this study, out of the 38 identified candidate transcripts of photosystem proteins, nine genes were upregulated by increasing CO_2_ levels, including four PSI proteins (ycf4, subunit III, reaction center subunit XI, and chlorophyll A apoprotein) and five PSII proteins (D1, D2, CP43, CP47, and PsbP). These findings suggest the importance of CO_2_ in enhancing the quantum efficiency of photosystems I and II [78], albeit the photosynthetic responses of seaweeds to increase in CO_2_ levels are still mostly unknown.

Light-regulated gene expression mediated by photoreceptors was studied previously in brown alga *S. japonica* in which the expression of photoreceptor genes was found triggered by different wavelengths of light, i.e., BL, RL, and WL [7]. However, in the present study, the phytochrome (one of the photoreceptors) gene was observed to be transcribed only under WL but not under other light of other wavelengths, suggesting that other characteristics of light may regulate the expression of phytochrome genes in *K. alvarezii*. On the other hand, the gene for cryptochrome (another photoreceptor) was positively expressed under all wavelengths of light and an increased concentration of CO_2_ except under RL treatment. Danon et al. [79] and Lopez et al. [80] reported that in plants, cryptochrome is involved in the light-dependent gene expression, mainly affecting the expression of genes involved in the responses to biotic/abiotic stresses and regulation of photosynthesis. Compared to BL, a higher expression of the cryptochrome gene under GL may be due to the discrete effects of this wavelength on plant biochemistry, i.e., physiological and developmental processes via cryptochrome-dependent and cryptochrome-independent means [81].

The identification of key enzymes involved in both the reductive pentose phosphate cycle (C_3_) and phosphoenolpyruvate carboxykinase (PCK)-type C_4_-dicarboxylic acid cycle indicates the possibility of the existence of both photosynthetic pathways (C_3_ and C_4_) in *K. alvarezii*. The levels of most of the enzymes involved in the C_3_-cycle, such as glyceraldehyde 3-phosphate dehydrogenase, fructose-bisphosphate aldolase, and phosphoglycerate kinase, are upregulated under elevated CO_2_, suggesting that CO_2_ enrichment may alter carbon metabolism from C_4_ to C_3_ in *K. alvarezii*, which is similar to that reported in other marine algae [22,82,83]. Alternatively, an increase in CO_2_ levels induces stomatal closure in terrestrial C_4_ plants, which reduces the loss of water and improves water use efficiency. C_4_ plants demonstrate drastically reduced rates of photorespiration because CO_2_ is concentrated at the active site of ribulose-1,5-bisphosphate carboxylase and outcompetes molecular oxygen, leading to higher photosynthetic efficiency [84]. However, the underlying photosynthetic mechanisms of *K. alvarezii* in elevated CO_2_ concentrations are largely unknown; besides, the process of gaseous diffusion is not regulated due to the absence of stomata [85,86]. Furthermore, the involvement of genes in the photosynthetic pathway in alternative physiological processes such as CO_2_ regulation remains to be elucidated, as in the case of carbonic anhydrase [87] and phosphoenolpyruvate carboxykinase [88]. Therefore, this response of red algae to increasing CO_2_ warrants further research.

## 4. Materials and Methods

### 4.1. Seaweed Materials

Seedlings of *K. alvarezii* (var. *tambalang* ‘giant’) were obtained from the Biotechnology Research Institute, Universiti Malaysia Sabah. The seaweed materials were collected from Pulau Sebangkat (4°33′31″ N, 118°39′49″ E), Semporna, Sabah and cultured under laboratory conditions following earlier published protocol [89]. The cultures were maintained in a growth chamber at 25.0 ± 1.0 °C and 18/6 h (light/dark) photoperiod with a light intensity of 75 μmol photons m^−2^ s^−1^. The experimental samples were selected from healthy, disease-free explants and cut to a total length of 2–3 cm from the algal tips. Three replicates, each consisting of five algal tips from different plants were used for each experimental condition. The algal tips were cultured in a Fernbach culture flask with 800 mL artificial seawater (Fluval marine salt, 36 g L^−1^, salinity 31.4 ppt) enriched with 50% Provasoli’s enriched seawater.

### 4.2. Culture Conditions

For light treatment, the cultures were placed under 75 μmol photons m^−2^ s^−1^ WL, BL (wavelength = 450–490 nm), GL (wavelength = 520–560 nm), and RL (wavelength = 635–700 nm) separately. The source of light were light-emitting diodes, and the detected irradiances were measured with a digital light meter (Model 5202, Kyoritsu, Japan). For CO_2_ treatment, compressed CO_2_ (500 mL/min flow rate; filtered with 0.2 µm sterile Acrovent filter) was added daily to WL-illuminated cultures for 30 min during the light cycle [90,91,92]. The measured concentrations of CO_2_ using a multiparameter meter (Orion, Thermo Scientific) were 994.9 ± 13.5 ppm in CO_2_-treated and 398.7 ± 11.1 ppm in untreated seawater media. CO_2_ was only supplemented during the light cycle to avoid acidification of the culture media, and pH was recorded using an electrochemistry portable meter (Orion, Thermo Scientific); it was determined to be 7.42 and 8.16 of the CO_2_-treated and untreated seawater, respectively. All the cultures (three biological replicates for each treatment and five seedlings per replicate) were grown at 25.0 ± 1.0 °C with an 18/6 h (light/dark) photoperiod. After culturing for 14 days, *K. alvarezii* seedlings were collected and pooled according to the respective treatments for RNA extraction.

### 4.3. RNA Isolation

Total RNA was extracted using the QIAGEN RNeasy Plant Mini Kit (QIAGEN, Germany) following the manufacturer’s protocol. Before extraction, the samples were washed with diethylpyrocarbonate-treated water and then rapidly frozen with liquid nitrogen and ground to a fine powder using an RNase-free, chilled mortar and pestle. RNA samples with A260/280 ratio between 1.8 and 2.0, A260/230 ratio between 1.8 and 2.1, and RNA integrity number (RIN) of 8 and above were used for library construction.

### 4.4. Preparation of cDNA Library

Five cDNA libraries were generated for each RNA sample of different culture treatments, i.e., BL, GL, RL, WL, and CO_2_, using the NEBNext Ultra RNA Library Prep Kit for Illumina (New England Biolabs, UK) following the manufacturer’s protocol. Then, the mRNA was purified from total RNA using poly-T oligo-attached magnetic beads and sheared into short fragments of about 200 bp length. The first-strand cDNAs were synthesized from the cleaved short RNA fragments using random hexamer-primers (NEB, UK) and reverse transcriptase (NEB, UK). The second strand cDNAs were synthesized using DNA polymerase I (NEB, UK) and RNase-H (NEB, UK). The purified double-stranded cDNA was subjected to end repair and NEBNext adaptor ligation. Then, the resulting libraries were loaded onto a single lane of the flow cell and sequenced for 209 cycles on the Illumina HiSeq 2000 platform (Illumina, San Diego, CA, USA) at the Malaysian Genomics Resource Centre. The high-quality reads were deposited in the NCBI SRA.

### 4.5. Quality Control and De Novo Transcriptome Assembly

Sequencing reads from the Illumina sequencer were exported in FASTQ format with the corresponding Phred quality scores. The quality of the sequencing raw reads was first evaluated with FastQC v0.11.2 (www.bioinformatics.babraham.ac.uk/projects/fastqc, accessed on 28 April 2021) and then filtered with Trimmomatic [93] before assembly by (i) removing the sequencing adaptor sequences, (ii) filtering the reads containing ambiguous nucleotides, and (iii) removing the reads having more than 10% bases below Q20 sequencing quality. The obtained filtered reads were subjected to transcriptome de novo assembly using Trinity software version 2.2.0 [94]. Briefly, the reads were assembled using Inchworm Program by searching for paths in a 25 bp *k*-mer graph and resulted in a collection of linear contigs. Next, the reads were clustered using the Chrysalis Program with a certain length of overlap, and then, a de Bruijn graph was constructed for each cluster. Finally, the Butterfly Program was used to reconstruct plausible full-length, linear transcripts by reconciling the individual de Bruijn graphs generated by Chrysalis. After generating the final assembly, the redundant sequences were removed using the TIGR Gene Indices clustering tools software [95].

### 4.6. Transcriptome Analysis

The assembled sequences were evaluated for similarity by using the Basic Local Alignment Search Tool (BLAST) translated nucleotide query to protein database (blastx) against the UniProt protein database [96] and Kyoto Encyclopaedia of Genes and Genomes (KEGG) database [97,98], respectively, and the cutoff was set at 10^−5^ *E*-value. Then, the sequences were functionally annotated using the Blast2GO [99] software to obtain Gene Ontology (GO) annotation before further classifying using the WEGO (Web Gene Ontology Annotation Plot) software [100].

### 4.7. Identification of DEGs

The RNA-Seq by Expectation-Maximization (RSEM) software version 1.2.11 was used to estimate transcripts abundance [101], and the empirical analysis of digital gene expression in R (edgeR) software version 3.8.6 in the Bioconductor package was used to determine differential gene expression between the two groups [102]. The reads were normalized using the TMM (trimmed mean of M)-values method [103]. To analyze differential expression, the transcripts with an adjusted *p*-value < 1 × 10^−3^ and log_2_ fold change ≥ 2 were extracted and clustered according to their patterns of differential expression across the sample. All the DEGs were mapped to the terms in the KEGG database and searched for significantly enriched KEGG pathways in comparison to the whole transcriptome background.

### 4.8. Quantitative Real-Time PCR Analysis

The cDNA was synthesized with the QuantiTect Reverse Transcription kit (QIAGEN, Germany). The 18S rDNA gene (GenBank accession number U25437) was chosen as an internal control for the normalization of real-time PCR data. Primers for amplification of targeted genes, i.e., light-harvesting complex A (LHCA), Von Willebrand factor type A (VWA), phosphoserine aminotransferase (PSAT) genes, and the 18S rDNA gene (Table 3) were designed using Primer3 software [104] aiming for amplicon lengths of 80–200 bp, a GC content of 40–60%, a primer length of 18–30 bp, and a primer melting temperature of 60–65 °C. Primers were assessed for melting temperature, and the formation of hairpins and primer dimers using the web-based tool Beacon Designer (http://free.premierbiosoft.com, accessed on 28 April 2021). Real-time quantitative PCR was performed in a 20 µL reaction volume containing QuantiNova SYBR green master mix (QIAGEN, Germany) on the Eco Real-Time PCR System (Illumina), and the cycling conditions were 95 °C for 2 min, 40 cycles at 95 °C for 5 s, and 60 °C for 10 s. The PCR reactions, together with negative control, were carried out in triplicates, and data were analyzed using the 2^−ΔΔCT^ method [105].

## Figures and Tables

**Table 1:** The top ten pathways identified in the *Kappaphycus alvarezii* transcriptome.

Pathway	Number of Transcripts	Pathway ID
Metabolic pathways	814	Ko01100
Biosynthesis of secondary metabolites	337	Ko01110
Biosynthesis of amino acids	131	Ko01230
Carbon metabolism	117	Ko01200
Ribosome	111	Ko03010
Purine metabolism	95	Ko00230
Oxidative phosphorylation	94	Ko00190
Pyrimidine metabolism	73	Ko00240
RNA transport	70	Ko03013
Spliceosome	65	Ko03040

**Table 2:** Transcripts involved in inorganic carbon fixation that were identified in *Kappaphycus alvarezii*.

Pathway	Enzyme	Number of Transcripts
C_4_-CCM	Carbonic anhydrase (CA)	23
	Alpha-carbonic anhydrase (α-CA)	2
	Beta-carbonic anhydrase (β-CA)	1
	Gamma-carbonic anhydrase (γ-CA)	3
	Malate dehydrogenase (MDH)	3
	Malate dehydrogenase (MDH2)	1
	Malate dehydrogenase (oxaloacetate-decarboxylating) (NADP+)	7
	Aspartate aminotransferase, cytoplasmic (GOT1)	3
	Aspartate aminotransferase, mitochondrial (GOT2)	3
	Pyruvate orthophosphate dikinase (PPDK)	5
	Phosphoenolpyruvate carboxylase (PEPC)	6
	Phosphoenolpyruvate carboxykinase (PEPCK)	1
C_3_	Glyceraldehyde 3-phosphate dehydrogenase (GAPDH)	10
	Transketolase	10
	Phosphoribulokinase (PRK)	1
	Phosphoglycerate kinase (PGK)	6
	Sedoheptulose-bisphosphatase (SBPase)	2
	Ribulose-bisphosphate carboxylase large chain (rbcL)	3
	Fructose-bisphosphate aldolase, class I (ALDO)	13
	Fructose-bisphosphate aldolase, class II (FBA)	6
	Ribulose-phosphate 3-epimerase (rpe)	6
	Triosephosphate isomerase (TIM)	6
	Ribose 5-phosphate isomerase A (rpiA)	2
	Fructose-1,6-bisphosphatase I (FBP)	6

**Table 3:** Selected genes and list of primers used for real-time PCR analysis.

Gene Name ^1^	Forward Primer (5′-3′)	Reverse Primer (3′-5′)	Amplicon Length (bp)	Annealing Temperature (°C)
LHCA	GTGCAAACACGCGCACCAGAGATGG	CAGCTCCCTTTCAACGCACAACAGCG	138	60
VWA	AGACTGCGTTCCTATCACCGCCAGC	CCGGCAGCAACATTGGGTCATCAGC	141	60
PSAT	ATTCGGTCGAGAGTGCGTGCAAGGG	ACAGAGGCGCAGAAGAGAGGGTTGC	195	60
18S	CTGCCTTCCTAGACGGACTG	CGAGCGGATTTAGAGATTGG	159	60

^1^ LHCA, light-harvesting complexes; VWA, Von Willebrand factor type A; PSAT, phosphoserine aminotransferase; 18S, 18S rRNA gene for normalization.

**Table 4:** Analysis of differential expression at transcript level.

Condition 1 ^1^	Condition 2 ^1^	All transcripts (No Filters)			Significant DEG (*p*-adj < 1 × 10^−10^ & log_2_ Fold Change of 2)		
		Total	Upregulation ^2^	Downregulation ^3^	Total	Upregulation ^2^	Downregulation ^3^
WL	BL	40,472	11,621	28,851	33	5	28
WL	GL	49,189	21,293	27,896	30	4	26
WL	RL	40,340	11,570	28,770	32	0	32
WL	CO_2_	41,831	12,211	29,620	138	74	64
BL	GL	31,042	20,000	11,042	10	9	1
BL	RL	21,571	10,331	11,240	30	12	18
GL	RL	31,673	11,427	20,246	59	24	35
BL	WL + GL + RL	52,342	40,459	11,883	14	11	3
GL	WL + BL + RL	52,342	30,861	21,481	332	25	307
RL	WL + BL + GL	52,342	40,323	12,019	49	31	18
WL	BL + GL + RL	52,342	21,730	30,612	582	4	578
BL + GL	RL + WL	52,342	29,643	22,699	2	2	0
BL + RL	GL + WL	52,342	38,999	13,343	0	0	0
GL + RL	BL + WL	52,342	31,097	20,435	0	0	0

^1^ WL, white light; BL, blue light; GL, green light; RL, red light. ^2^ Upregulation of condition 2 over condition 1. ^3^ Downregulation of condition 2 over condition 1.

## Data Availability

The high-quality reads were deposited in the National Center for biotechnology Information (NCBI) Sequence Read Archive (SRA). The raw sequenced read was deposited at the NCBI SRA database with the accession number of SRR2757332 (GL), SRR2757333 (BL), SRR2757334 (CO_2_-treated), SRR2757335 (WL) and SRR2757337 (RL). The deposited data can be accessed at [https://www.ncbi.nlm.nih.gov/sra/SRX1360503[accn], accessed on 28 April 2021.
